# Supplementary material for: Plasma Extracellular Vesicle Characteristics as Biomarkers of Resectability and Radicality of Surgical Resection in Pancreatic Cancer—A Prospective Cohort Study
Source: Cancers (Basel). 2023 Jan 18;15(3):605. doi: 10.3390/cancers15030605 (PMC9913838; doi:10.3390/cancers15030605)
Supplement: Supplementary file 1 [file cancers-15-00605-s001.zip › cancers-2107022-supplementary.pdf]

Table S1. Particles per frame for NTA measurements of plasma sEV samples.

	**Study patients**	**w/o resection**	**With resection**
	**Median PPF (25-75%)**	**Median PPF (25-75%)**	**Median PPF (25-75%)**
Before surgery (N=82: 33 w/o resection, 49 with resection)	20.48 (15.29-26.93)	18.65 (14.65-24.80)	22.15 (16.00-27.50)
After one month (N=53: 16 w/o resection, 37 with resection)	23.75 (16.45-33.35)	21.23 (15.70-33.54)	25.70 (18.20-32.70)
After six months (N=43: 9 w/o resection, 34 with resection) 35	24.70 (19.35-32.65)	20.70 (16.00-24.00)	25.25 (19.80-33.60)
After 12 months (N=29: 1 w/o resection, 28 with resection)	21.70 (17.65-31.15)	32 *	21.65 (17.65-29.80)

PPF: particles per frame; *: After 12 months, data for a single patient in the group without resection was available

Table S2. Small EV characteristics one month after surgery compared to characteristics at surgery.

**EV characteristics**	**p-Value***	
**Study patients (N=52)**		
Concentration (N*10^9/mL)	0.483	Rises in 28, falls in 24 patients
Mean diameter (nm)	0.018	Rises in 32, falls in 20 patients
**w/o resection (N=16)**		
Concentration (N*10^9/mL)	0.352	Rises in 10, falls in 6 patients
Mean diameter (nm)	0.796	Rises in 6, falls in 10 patients
**With resection (N=36)**		
Concentration (N*10^9/mL)	0.814	Rises in 18, falls in 18 patients
Mean diameter (nm)	0.014	Rises in 26, falls in 10 patients
**R0 resection (N=22)**		
Concentration (N*10^9/mL)	0.227	Rises in 8, falls in 14 patients
Mean diameter (nm)	0.010	Rises in 17, falls in 5 patients
**R1 or R2 resection (N=14)**		
Concentration (N*10^9/mL)	0.041	Rises in 10, falls in 4 patients
Mean diameter (nm)	0.397	Rises in 9, falls in 5 patients

Table S3. Characteristics of sEV at different time intervals.

		**R0 resection (including border <1 mm)**	**R1 or R2 resection**	
	**Small EV characteristics**	**Median (25-75%)**	**Median (25-75%)**	**p-Value**
Before surgery (N=48: 31 R0, 17 R1+R2)	Concentration (N*10^9/mL)	2.68 (1.98-3.45)	1.85 (1.45-2.46)	0.014
	Mean diameter (nm)	179.5 (171.8-186.1)	182.7 (166.7-194)	0.497
After one month (N=37: 23 R0, 14 R1+R2)	Concentration (N*10^9/mL)	2.29 (1.83-2.88)	2.56 (1.59-3.6)	0.676
	Mean diameter (nm)	183.7 (176.7-194.1)	186.2 (172.5-199.6)	0.988
After six months (N=34: 21 R0, 13 R1+R2)	Concentration (N*10^9/mL)	2.98 (1.92-3.31)	2.68 (1.67-3.5)	0.780
	Mean diameter (nm)	181.1 (177.5-188.7)	181.4 (168.8-194.9)	1.000
After 12 months (N=28: 20 R0, 8 R1+R2)	Concentration (N*10^9/mL)	2.4 (1.58-3.27)	3.08 (1.97-5.75)	0.199
	Mean diameter (nm)	173.2 (163.8-186.6)	191.3 (178-198.1)	0.079

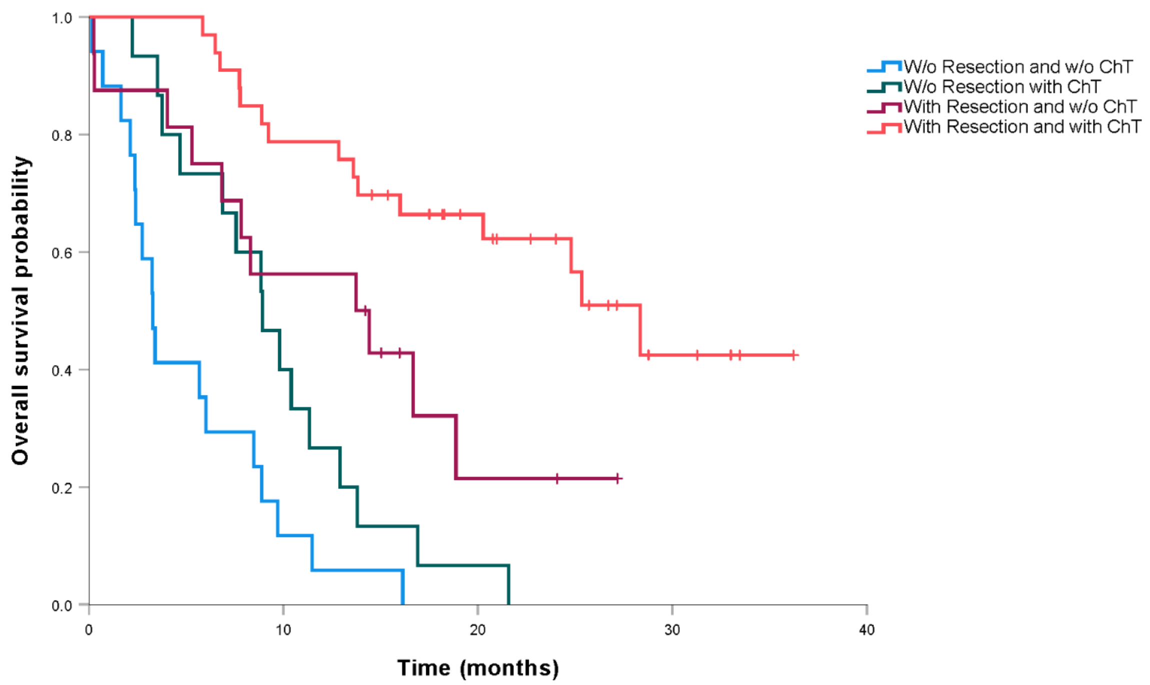

Figure S1. OS of patients with PDAC according to treatment modality. Patients without surgical resection and without chemotherapy demonstrated the worst OS, while patients with surgical resection and adjuvant chemotherapy had the best OS; patients without resection with only chemotherapy and those with only resection and no chemotherapy are positioned in between. w/o: without; ChT: chemotherapy; censored patients are presented with a vertical line.
